# New Osmylopsychopid Taxa from the Middle Jurassic of Northeastern China (Neuroptera: Osmylopsychopidae) †

**DOI:** 10.3390/insects14050484

**Published:** 2023-05-21

**Authors:** Xiaotian Liu, Dong Ren, Chungkun Shih, Yongjie Wang

**Affiliations:** 1 College of Life Sciences, Capital Normal University, Beijing 100048, China; liuxiaotiancnu2022@163.com (X.L.); rendong@cnu.edu.cn (D.R.); chungkun.shih@gmail.com (C.S.); 2 Guangdong Key Laboratory of Animal Conservation and Resource Utilization, Guangdong Public Laboratory of Wild Animal Conservation and Utilization, Institute of Zoology, Guangdong Academy of Sciences, Guangzhou 510260, China; 3 Department of Paleobiology, National Museum of Natural History, Smithsonian Institution, Washington, DC 20013-7012, USA

**Keywords:** Daohugou, insect fossil, Jiulongshan Formation

## Abstract

**Simple Summary:**

Osmylopsychopidae, an extinct family of Neuroptera, is closely related to the extant Psychopsidae. The sporadic records of this family and limited preservation of fossil specimens have hindered the understanding of the evolution of Osmylopsychopidae. In this paper, one new genus with four new species of Osmylopsychopidae are described from the Middle Jurassic Jiulongshan Formation of northeastern China. These newly discovered osmylopsychopids with a forewing length of ca. 10 mm (implying small body size), clearly distinguished from the traditional sense of Osmylopsychopidae, suggest that Osmylopsychopidae possibly underwent a significant diversification during the Mesozoic.

**Abstract:**

One new genus with four new species sharing the similar character of a forewing length of ca. 10 mm, namely, *Minipsychops spasulus* gen. et sp. nov., *Minipsychops polychotomus* sp. nov., *Minipsychops densus* sp. nov., and *Minipsychops unicus* sp. nov., are described from the Middle Jurassic Jiulongshan Formation of Daohugou, Inner Mongolia in China. These new insects can be attributed to Osmylopsychopidae for their distinctive configuration of costal space and the venations of RP1 and Cu. Compared with the typically medium to large body sizes of known osmylopsychopids, these new Middle Jurassic taxa of a particularly miniaturized lineage not only enrich the species diversity of Osmylopsychopidae, but also enhance our understanding of the historical evolution of these poorly known lacewings.

## 1. Introduction

Osmylopsychopidae is a mysterious extinct family of Neuroptera, represented by 16 genera and 23 species hitherto that are mainly distributed in Eastern Asia (China, Japan), Australia, and Europe [1,2,3,4,5,6,7,8,9,10]. In general, Osmylopsychopidae is believed to be the sister group to the extant Psychopsidae for their highly morphological homologue [11]. However, the controversies of the familial status of Osmylopsychopidae were raised by some authors who proposed to synonymize Osmylopsychopidae with Psychopsidae owing to the lack of autapomorphies of Osmylopsychopidae [12]. In fact, many authors attempted to make a taxonomic diagnosis for them [8,12,13], but the problem still seems to be far from being resolved. Before the final resolution of these two families, we still treat Osmylopsychopidae as a separated family that is characterized by the following combination of characteristics based on the summaries from previous works [8,12]: medium to large body size; costal space of the forewing significantly broadened in base and gradually narrowed to the end; and RP1 deeply forked with many branches.

To date, the oldest representatives of Osmylopsychopidae were documented, respectively, from the Late Triassic (Carnian) of Queensland, Australia, and France [3,4,9,14,15,16]. Although these types of specimens are incompletely preserved, it is concluded that all Triassic osmylopsychopids from the southern hemisphere belong to the large insects whose forewing length is more than 40 mm, inferred from the preserved fossil specimens [3,4]. Noticeably, the only Triassic osmylopsychopid from the northern hemisphere distinctly belongs to a small insect whose forewing length is only 13 mm [1]. In the Jurassic, abundant fossils of Osmylopsychopidae from Asia were documented mainly from two renowned localities, i.e., the Early Jurassic and Middle Jurassic of Kyrgyzstan and the Middle Jurassic of Daohugou [2,8]. Hitherto, four genera and four species of Osmylopsychopidae from Kyrgyzstan have been reported, of which most species have a medium body size (forewing lengths ranged 20–30 mm) except for *Oligophlebiopsis biramosa*, Khramov and Makarkin, 2015 (hind wing length of less than 10 mm) [2]. The Daohugou osmylopsychopids are distinctly more diverse, consisting of 10 species in 5 genera [8]. Similar to the Kyrgyzstan lineages, the Daohugou osmylopsychopids also have a medium to large body size [8]. In the Cretaceous, the number of species of Osmylopsychopidae was significantly decreased, and only one Cretaceous species, i.e., *Undulopsychopsis alexi*, Peng, Makarkin, and Wang, 2015, was described with a forewing length of 21.5 mm [7]. Considering the condition of historical diversity, the Osmylopsychopidae unequivocally underwent a rapid diversification during the Jurassic, which was characteristic of the high species diversity and abundant morphological specializations. Herein, one new genus with four new species of small osmylopsychopids (with forewing lengths of about 10 mm) from the Middle Jurassic in Daohugou are described for the first time. The descriptions of these new species enriched the species diversity of the Jurassic Osmylopsychopidae and provided new information for exploring the early evolution of Osmylopsychopidae.

## 2. Materials and Methods

These new fossil specimens were collected from the Middle Jurassic Jiulongshan Formation at Daohugou Village, Shantou Township, Ningcheng County, Inner Mongolia, China. In the past 20 years, many paleontologists have successively found highly abundant and well-preserved fossil specimens in Daohugou, including gymnosperm fossils (such as ginkgo, conifers, cycads, etc.), vertebrate fossils (such as fish, amphibians, reptiles, primitive birds, mammals, etc.) and insect fossils. Therefore, the Daohugou area, with a humid and warm climate and abundant vegetation, is currently one of the important paleontological discoveries [17,18,19,20].

All fossil specimens of this study are deposited in the Key Laboratory of Insect Evolution and Environmental Changes, College of Life Sciences, Capital Normal University, Beijing (CNUB; Dong Ren, Curator). The specimens were observed and photographed by using a stereoscopic microscope Nikon SMZ25 with an attached Nikon DS-Ri2 digital camera system. A more detailed method for taking photos of the specimens is to continuously adjust the angle of the two sets of light projectors equipped with the stereoscopic microscope to present the full view of the specimen more clearly. For most of the specimens in this article, we mainly placed a set of illuminators on one side of the specimen to allow the light source to be emitted from one side, as parallel as possible to the specimen plane, which is conducive to reflecting the three-dimensional sense of the specimen and making the concave and convex sense of the wing veins more obvious. Based on the obvious unevenness of some specimens, the superposition function of the Nikon DS-Ri2 digital camera system itself can be used to integrate photos of specimens from different planes to make every part of the specimen as clear as possible. In addition, the method of dropping alcohol was used to observe the details of some specimens. Adding alcohol to the specimens can make the light scatter, reduce the reflection of light on the specimens, enhance the contrast of fossil specimen imaging, and make the specimens clearer.

The venational terminology follows Breitkreuz et al. [21]. The wing vein abbreviations are as follows: C, costa; Sc, subcosta; ScA, subcosta anterior; ScP, subcosta posterior; R, radius; RA, radius anterior; RP, radius posterior; RP1, the first branch of the radius posterior; M, media; MA, media anterior; MP, media posterior; Cu, cubitus; CuA, cubitus anterior; CuP, cubitus posterior; A, anal vein; A1, the first anterior anal vein; A2, the second anterior anal vein.

## 3. Results

Systematic paleontology.

Order Neuroptera Linnaeus, 1758.

Superfamily Psychopsoidea Handlirsch, 1906.

Family Osmylopsychopidae Martynova, 1949.

Genus *Minipsychops* Liu, Ren, Shih, and Wang gen. nov.

Type species. *Minipsychops sparsulus* Liu, Ren, Shih, and Wang sp. nov.

Species included. *Minipsychops sparsulus* sp. nov.; *Minipsychops polychotomus* sp. nov.; *Minipsychops densus* sp. nov.; *Minipsychops unicus* sp. nov.

Diagnosis. The forewing is easily separated from those of the other genera of Osmylopsychopidae by the combination of the following character states: (1) the forewing length of about 10 mm; (2) RP1 deeply forked 1–3 times; (3) ScP and RA parallel and fused distally; (4) M forked near the first branched point of the RP1; (5) Cu forked near the base of the wing; (6) CuA forked near the middle part of this vein; and (7) CuP forked near the origin of the CuP.

Etymology. From the Greek prefix *mini*- (meaning “small”), and -*psychops*, a traditional generic ending of Osmylopsychopidae, referring to the small body size of the new genus. Gender masculine.

**Figure 1:**
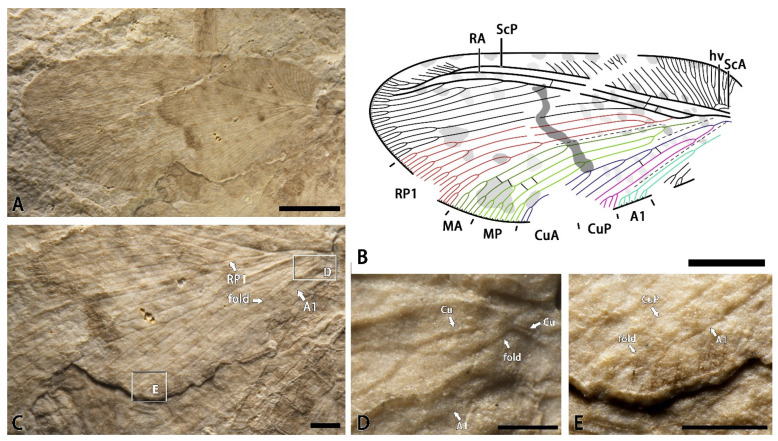
*Minipsychops sparsulus* gen. et sp. nov.; (**A**) photograph of holotype (CNU-NEU-NN-2022001); (**B**) line drawing of forewing venation of holotype; (**C**) photograph of part of holotype; (**D**) photograph of preserved forewing base details of holotype (as located on Figure 1C); (**E**) photograph of preserved forewing details with the branches near the distal part of the vannal region (as located on Figure 1C). Scale bars: (**A**,**B**) for 2 mm; (**C**,**E**) for 0.5 mm; (**D**) for 0.25 mm.

**Figure 2:**
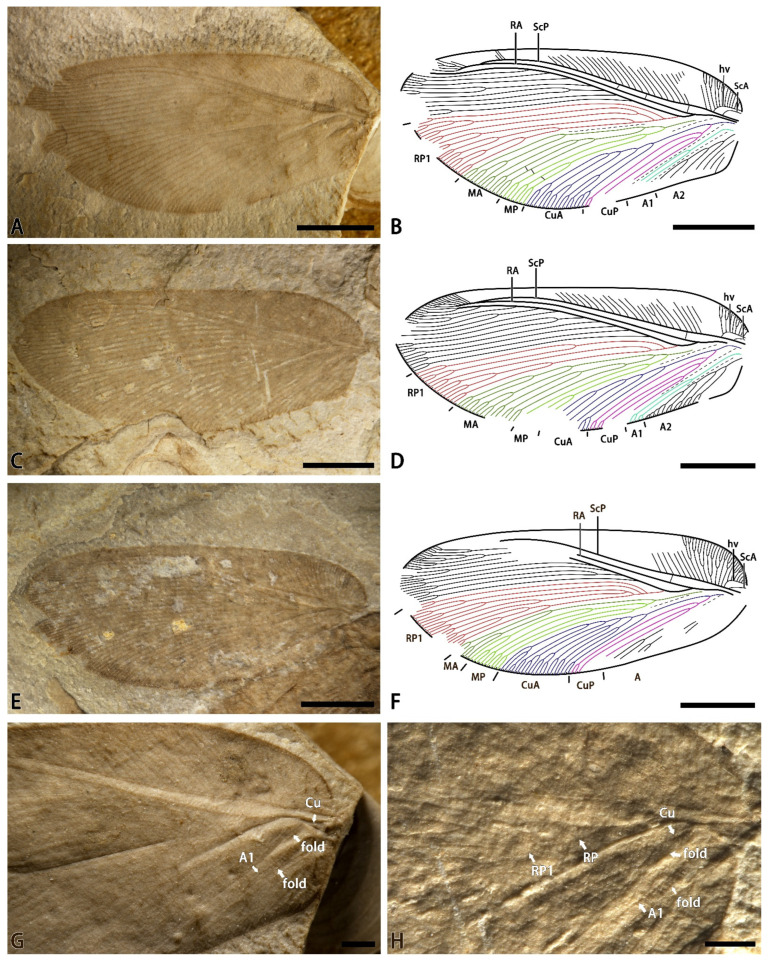
*Minipsychops polychotomus* sp. nov.; (**A**) photograph of holotype (CNU-NEU-NN-2022002); (**B**) line drawing of the forewing venation of holotype (CNU-NEU-NN-2022002); (**C**) photograph of paratype (CNU-NEU-NN-2022003); (**D**) line drawing of the forewing venation of paratype (CNU-NEU-NN-2022003); (**E**) photograph of paratype (CNU-NEU-NN-2022004); (**F**) line drawing of the forewing venation of paratype (CNU-NEU-NN-2022004); (**G**) photograph of preserved forewing base of holotype; (**H**) photograph of preserved forewing base of paratype. Scale bars: (**A**–**F**) for 2 mm; (**G**,**H**) for 0.5 mm.

Remarks. *Minipsychops* gen. nov. belongs to the Osmylopsychopidae based on the following characteristics: (1) costal space sharply narrowed from the base to the end, (2) forewing nearly oblong, and (3) RP1 deeply forked more than once. This new genus is easily distinguished from most other genera of Osmylopsychopidae by its shorter forewing length, implying a smaller body size (vs. a commonly medium to large body size). Additionally, two osmylopsychopid species, i.e., *Osmopsychops radialis*, Ellenberger, Laurentiaux and Ricour, 1952, from France in the Late Triassic and *Oligophlebiopsis biramosa*, Khramov and Makarkin, 2015, from Kyrgyzstan in the early Middle Jurassic, have similar smaller body sizes as the new genus. The new genus can be easily distinguished from *Osmopsychops* by the position of Sc and RA and the branched pattern of M and Cu [1: Figure 1]. Because the type specimen of *O. biramosa* is preserved as the hind wing [2: Figure 2], the new genus cannot be fully compared with it. However, considering the general homogeneity of venation within the forewing and hind wing, the new genus is putatively separated from *O. biramosa* by the more complicated MA and MP branching (vs. simple distal branching in *O. biramosa*).

*Minipsychops sparsulus* Liu, Ren, Shih, and Wang gen et sp. nov.

Figure 1.

urn:lsid:zoobank.org:act:45844432-1DC3-4D43-919F-310493D3E45F

Material. Holotype: CNU-NEU-NN-2022001, a nearly complete forewing with the poorly preserved proximal part (Figure 1A,B).

Etymology. From the Latin *sparsulus* (meaning “sparse”), referring to the sparse venation of the new species.

Locality and horizon. Latest Middle Jurassic (late Callovian), Jiulongshan Formation; Daohugou Village, Shantou Township, Ningcheng County, Inner Mongolia, China.

Diagnosis. RP1 deeply forked two times with two upward branches; =MA and MP deeply branched with simple primary sub-branches; the CuA pectinately branched with four primary branches; the CuP deeply dichotomously forked once; and presence of colored markings on the forewing.

Description. Forewing nearly triangular, 9.8 mm long, and 4.4 mm wide. Trichosors present along the entire margin. Costal space very broad basally, and distinctly narrowed toward the apex. Subcostal veinlet distinctly branched in the distal part. Humeral veinlets strongly recurrent with five sub-branches. ScA present. ScP and RA parallel and fused distally. Presence of one crossvein in the subcostal spaces base. RA simple for most of its length with few branches distally, and presence of three crossveins in the RA space. RP pectinately branched with eight primary branches; RP1 deeply forked with two upward primary branches. M forked near the first branched point of the RP1. MA dichotomously forked near the mid-point of this vein; the MP dichotomously forked with two primary branches near the branched point of the M. Cu forked at the base of the wing; CuA pectinately branched near mid-point of this vein with four primary branches; CuP dichotomously forked near the branched point of the Cu; and the terminal of the CuA poorly preserved, probably dichotomously forked distally. A1 dichotomously forked near the branched point of the CuP with two primary branches. A2 poorly preserved. Presence of several crossveins in the medial space and cubital space. Many fragmentary speckles scattered on the forewing, and a dark transversed irregular band present in the middle part of the forewing.

Remarks. The new species is mostly characterized by the presence of distinct markings on the forewing, especially a dark transverse stripe in the middle of the wing. Additionally, the M branches in the new species are distinctly different from other *Minipsychops* species.

*Minipsychops polychotomus* Liu, Ren, Shih and Wang sp. nov.

Figure 2.

urn:lsid:zoobank.org:act:086B0FC4-42C2-4B5E-BCB9-F695E4D24F5E

Material. Holotype: CNU-NEU-NN-2022002 (Figure 2A,B), a nearly complete forewing with a length of 8.8 mm and a width of 4.1 mm. Paratype: CNU-NEU-NN-2022003 (Figure 2C,D), a nearly complete forewing with a length of 9.6 mm and a width of 4.2 mm. Paratype: CNU-NEU-NN-2022004 (Figure 2E,F), a nearly complete forewing with a length of 9.7 mm and a width of 4.0 mm.

Etymology. From the Latin *polychotomus* (meaning “bifurcated”), referring to theRP1 with multiple forks.

Locality and horizon. Latest Middle Jurassic (late Callovian), Jiulongshan Formation; Daohugou Village, Shantou Township, Ningcheng County, Inner Mongolia, China.

Diagnosis. RP1 deeply forked with 2–3 upward branches; M branches with complicatedly multiple sub-branches; CuA pectinately forked; and CuP deeply dichotomously forked 2–3 times.

Description. Forewing nearly oblong. Trichosors present along the entire margin. Costal space very broad basally, distinctly narrowed toward the apex. Subcostal veinlets distinctly branched in the distal part. Humeral veinlets strongly recurrent, with 8–10 sub-branches. ScA present. ScP and RA approached each other toward the apex and fused distally. Presence of 1–3 crossveins in the subcostal spaces. RA simple for most of its length with few branches distally. RP pectinately branched with 8–10 primary branches; RP1 deeply forked with 2–3 upward primary branches. M forked near the first branched point of the RP1; MA dichotomously forked near the origin of the MA with several secondary sub-branches; and MP similarly deeply forked with two primary dichotomous branches. Cu forked at the base of the wing; CuA pectinately branched near the mid-point of the vein with five primary branches; and CuP dichotomously forked with several sub-branches near the origin of the CuP. A1 forked near the mid-point of this vein to give two primary branches. A2 well-developed, with four pectinated branches.

Remarks. Although the new species has a similar body size to the type species of *Minipsychops*, it can be easily distinguished from the type species by the absence of wing patterns and the distinctly complicated wing venation. In addition, this new species has three type specimens, of which RP1, M, and CuP exhibit remarkable intraspecific variations (Figure 2). In holotype CNU-NEU-NN-2022002 and paratype CNU-NEU-NN-2022003, they have a similar branching pattern of the M and CuA, but show minor differences in the number of RP1 and CuP (Figure 2B,D). The holotype CNU-NEU-NN-2022002 and paratype CNU-NEU-NN-2022004 exhibit similar RP1 branching and CuP, but the MA branching and CuA show some differences between them (Figure 2B,F). It is easily concluded that the differences among the type specimens show a distinctly continuous condition. Considering the complexity of venation in the genus, it is reasonable to treat these differences as the intraspecific variations rather than over-addressing the variations to set up too many new taxa that are defined by the continuous characteristics, which would possibly lead these taxa to be repeatedly difficult to identify. Thus, we proposed a broad approach for the continuous condition.

*Minipsychops densus* Liu, Ren, Shih, and Wang sp. nov.

Figure 3.

urn:lsid:zoobank.org:act:94114385-0047-4D8C-B80E-536959519A5B

**Figure 3:**
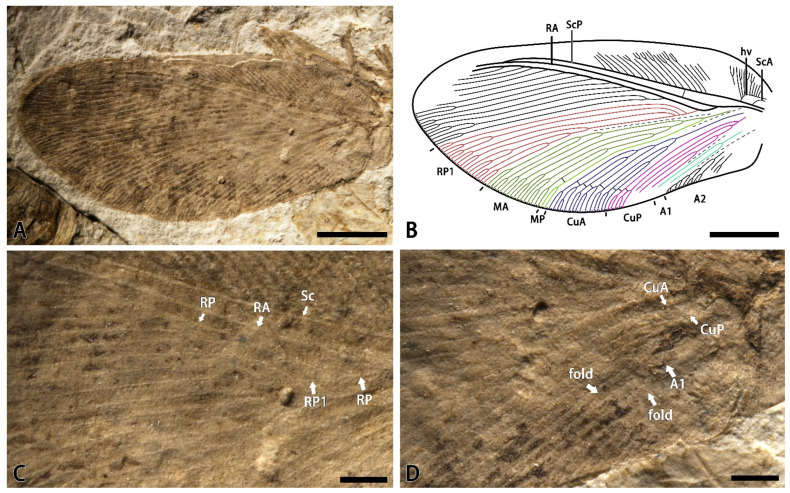
*Minipsychops densus* sp. nov.; (**A**) photograph of holotype (CNU-NEU-NN-2022005); (**B**) line drawing of the forewing venation of holotype (CNU-NEU-NN-2022005); (**C**) photograph of part of holotype; (**D**) photograph of preserved forewing base of holotype. Scale bars: (**A**,**B**) for 2 mm; (**C**,**D**) for 0.5 mm.

Material. Holotype: CNU-NEU-NN-2022005, a nearly complete forewing (Figure 3A,B).

Etymology. From the Latin *densus* (meaning “dense”), referring to the relatively numerous and densely spaced veins.

Locality and horizon. Latest Middle Jurassic (late Callovian), Jiulongshan Formation; Daohugou Village, Shantou Township, Ningcheng County, Inner Mongolia, China.

Diagnosis. RP densely branched with twelve primary branches; RP1 deeply forked three times with three upward branches; MP forked with two simple dichotomous branches in distal; CuA dichotomously branched with complicated sub-branches; and CuP pectinately forked.

Description. Forewing nearly triangular, 10.6 mm long, and 4.9 mm wide. Trichosors present along the entire margin. Costal space very broad basally, distinctly narrowed toward the apex. Subcostal veinlet distinctly branched in the distal part. Humeral veinlets strongly recurrent with at least ten sub-branches. ScA present. ScP and RA parallel and fused distally. Presence of at least one crossvein in the subcostal space. RA simple for most of its length with few branches distally. RP pectinately branched with 13 primary branches, and RP1 deeply forked with three upward primary branches. M forked near the first branched point of the RP1. MA dichotomously forked near the branched point of the M; MP simple for most of its length with few branches distally. Cu forked at the base of the wing; CuA dichotomously branched with complicated sub-branches near the mid-point of this vein, and CuP pectinately forked with four primary branches near the branched point of Cu. A1 dichotomously forked near the middle part of this vein with two primary branches. A2 pectinately forked distally. Presence a few crossveins on the distal part of the cubitus space, forming an incomplete and short graduate series. Presence of irregular dark traces, which may be wing patches, with gradually deepening color and no obvious boundary from the middle part of the forewing to the distal part of the wing.

Remarks. This new species is closely related to the *M. polychotomus* for sharing the similar RP1 branching; however, it can be easily separated from *M. polychotomus* by the following characteristics: more RP branches (more than 10 branches in the new species vs. 8–10 primary branches in *M. polychotomus*), simple MP branching (distally branched in the new species vs. deeply branched in *M. polychotomus*), dichotomously branched CuA (vs. pectinately branched in *M. polychotomus*), and more CuP sub-branches. In addition, this species seems to bear some dark markings on the distal part of the wing, which are also absent in *M. polychotomus*. Thus, we proposed a new species for this specimen.

It is noticed that *M. polychotomus* exhibits a high variation of venation due to the occurrence of several type specimens, but it has still not been determined whether *M. densus* also had the similar intra-specific variations. It is hoped that more specimens of *M. densus* will be found to enhance the knowledge of this species, which would also provide new insight to understand the species diversification of *Minipsychops.*

*Minipsychops unicus* Liu, Ren, Shih, and Wang sp. nov.

Figure 4.

urn:lsid:zoobank.org:act:FDEDDA31-35ED-4D0D-959F-76D0BAF3394D

**Figure 4:**
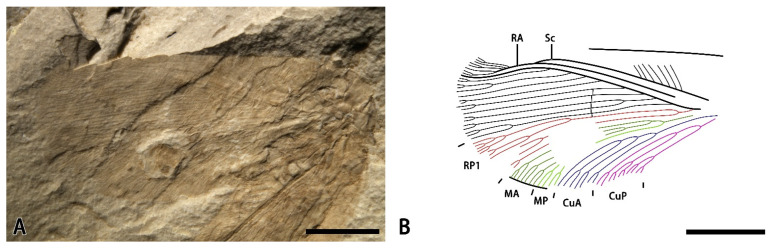
*Minipsychops unicus* sp. nov.; (**A**) photograph of holotype (CNU-NEU-NN-2022006); (**B**) line drawing of forewing venation of holotype (CNU-NEU-NN-2022006). Scale bars: (**A**,**B**) for 2 mm.

Material. Holotype: CNU-NEU-NN-2022006, an incomplete forewing with its outer margin poorly preserved (Figure 4A,B).

Etymology. From the Latin *unicus* (meaning “only one”), referring that the RP1 is deeply forked only once.

Locality and horizon. Latest Middle Jurassic (late Callovian), Jiulongshan Formation; Daohugou Village, Shantou Township, Ningcheng County, Inner Mongolia, China.

Diagnosis. RP1 deeply dichotomously forked; MA and MP simply branched; CuA forked with three simply pectinately branches, and CuP forked with two primary dichotomous branches.

Description. Forewing elongated, with an 8.4 mm preserved length (estimated length 9.1 mm), and 4.0 mm preserved width (estimated width 4.1 mm). Trichosors present along the entire margin. Costal space very broad basally, distinctly narrowed toward the apex. Presence of no humeral veinlets in the wing base because of the poor preservation. Subcostal veinlets with their terminal poorly preserved, and probably forked in the distal part. Sc and RA approached each other toward the apex and fused distally. Prensence of no crossveins in the subcostal spaces (probably due to poor preservation). RP pectinately branched with nine primary branches, and RP1 deeply forked with two primary branches. M forked slightly beyond the first branched point of the RP1, MA dichotomously forked near the separated point of the M, the forking of the MP invisible because of the poor preservation, and MP probably dichotomously forked distally. Cu dichotomously branched near the wing base; CuA pectinately branched near the mid-point of this vein with four primary branches, and CuP dichotomously forked near the branched point of the Cu, forming three primary dichotomous branches. A poorly preserved. Presence of five crossveins in the medial space at the middle part of the wing, forming a short and regular gradate series.

Remarks. This new species can be easily distinguished from other *Minipsychops* species by the number of RP1 (two dichotomous RP1 sub-branches in the new species vs. presence of at least two upward RP1 sub-branches in other species). Additionally, the M and CuA branches of the new species are relatively simple in contrast to other con-generic species.

## 4. Discussion

**Figure 5:**
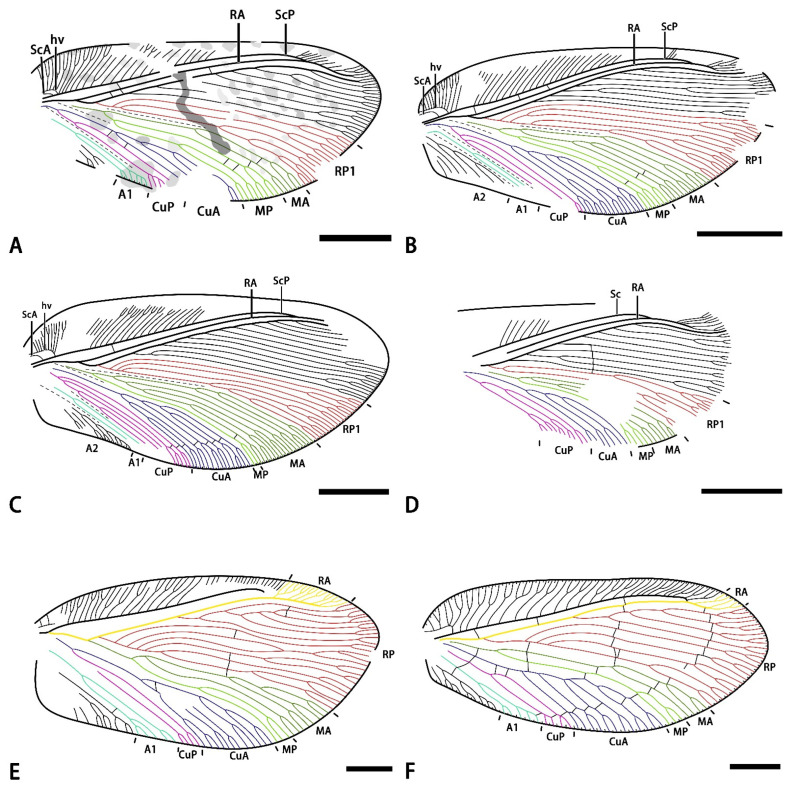
Comparison of venation of *Minipsychops* and two other fossil hemerobiid genera. (**A**) *Minipsychops sparsulus* gen. et sp. nov.; (**B**) *Minipsychops polychotomus* sp. nov.; (**C**) *Minipsychops densus* sp. nov.; (**D**) *Minipsychops unicus* sp. nov.; (**E**) the line redrawing of wing venation of *Promegalomus anomalus*, Panfilov in Dolin et al., 1980, by Panfilov et al. ([18], pic.91); (**F**) the line redrawing of wing venation of *Proneuronema minor*, Makarkin, Wedmann, and Weiterschan, 2016, by Makarkin et al. ([19], Figure 2A). Scale bars: (**A**–**D**) for 2 mm; (**E**,**F**) for 1 mm.

The *Minipsychops* species reported here and characterized by the smaller body size and complicated branching pattern of the RP1 superficially resemble some Mesozoic Hemerobiidae fossil, namely, *Promegalomus anomalus*, Panfilov in Dolin et al., 1980, from Kazakhstan, unearthed in the Jurassic and *Proneuronema minor*, Makarkin, Wedmann, and Weiterschan, 2016, from the Eocene Baltic Amber [22,23] (Figure 5E,F). Comparing these hemerobiid lineages, the *Minipsychops* species are distinctly distinguished by the origin position of the RP1. The RP1 of *Minipsychops* is clearly diverged from the stem of the RP branches and are parallel to the other RP branches, but the RP1 of the hemerobiids is normally separated from the RA instead of the RP stem (Figure 5). Considering the divergence position of the RP1 and the branching pattern of the RP branches, *Minipsychops* is more related to Osmylopsychopidae compared with Hemerobiidae.

The occurrence of *Minipsychops* unequivocally enhances our understanding to the diversification of osmylopsychopid during the Mesozoic. Within the Osmylopsychopids, the similar small sized osmylopsychopsids had been discovered from *Osmopsychops radialis*, Ellenberger, Laurentiaux, and Ricour, 1952, of the Triassic and *Oligophlebiopsis biramosa*, Khramov and Makarkin, 2015, of the Jurassic [1,2]. Based on their original descriptions, we found that *Minipsychops* exhibits some distinct differences with these two species, which are reflected in the RP1, M, and Cu [Figure 5A,D; 1: Figure 1; 2: Figure 2], implying their distant affinity. It also hints that the miniaturized osmylopsychopids had potentially independently evolved across different localities and times.

**Figure 6:**
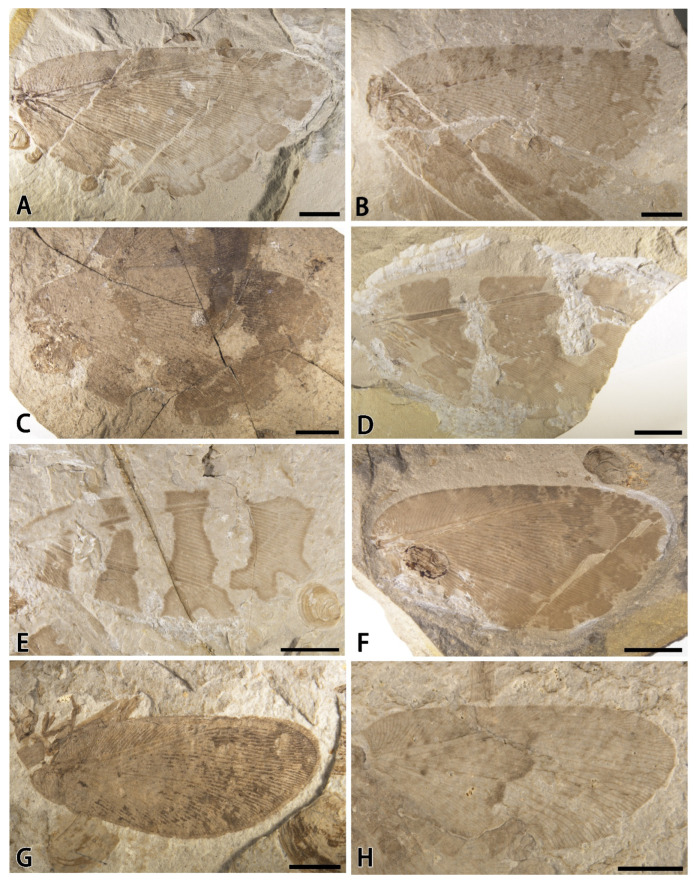
Representatives of Daohugou Osmylopsychopidae. (**A**) *Daopsychops dissectus*, Peng, Makarkin, and Ren, 2015, with forewing length about 38 mm; (**B**) *Eupypsychops ferox*, Peng, Makarkin, and Ren, 2015, with forewing length about 35 mm; (**C**) *Daopsychops cubitalis ferox*, Peng, Makarkin, and Ren, 2015, with forewing length about 32 mm; (**D**) *Daopsychops bifasciatus*, Peng, Makarkin and, Ren, 2015, with forewing length about 31 mm; (**E**) *Stenopteropsychops trifasciatus*, Peng, Makarkin, and Ren, 2015, with forewing length about 26 mm; (**F**) *Eupypsychops confinis*, Peng, Makarkin and, Ren, 2015,with forewing length about 24.5 mm; (**G**) *Minipsychops densus* gen. et sp. nov., with forewing length about 10.6 mm; (**H**) *Minipsychops sparsulus* sp. nov., with forewing length about 9.8 mm. Scale bars: (**A**–**F**) for 5 mm; (**G**,**H**) for 2 mm.

To date, ten species in five genera have been reported from Daohugou of the Middle Jurassic, representing the most diverse osmylopsychopids among the Mesozoic localities [8]. It is noteworthy that the Daohugou osmylopsychopids exhibited high morphological heterogeneity. Owing to the discovery of miniature *Minipsychops*, the body sizes of the Daohugou osmylopsychopids covered all ranges of known osmylopsychopids, of which the largest lineage is *Daopsychops dissectus*, Peng, Makarkin, and Ren, 2015, with a forewing length exceeding 38 mm. The high variations of body sizes among the Daohugou osmylopsychopids implied the potential diversity of their living habits during the Middle Jurassic period. In addition, the Daohugou osmylopsychopids were also characterized by the remarkable wing markings (Figure 6). In the general osmylopsychopids that have the medium to large body size, several types of wing patterns can be identified, e.g., membrane clouded with dark color with the distinct marginal hyaline patches in the outer margin (Figure 6A–C,F), and membrane bearing distinct transverse dark stripes (Figure 6D,E). Interestingly, the patterns of the wing markings within the Daohugou osmylopsychopids showed less generic stabilization. *Daopsychops*, the dominant genus of the Daohugou osmylopsychopids, has nearly all types of wing marking patterns of Osmylopsychopidae (Figure 6A,C,D). In the new genus, the wing markings of *Minipsychops sparsulus* represent a new type of wing marking pattern within Osmylopsychopidae, in which one dark sinuous transverse stripe is present in the middle of the forewing (Figure 6H), while the forewing of *M. densus* is entirely clothed with dark color and presents some black spots in the distal half (Figure 6G). Although we still do not know the biological functions of the wing markings among these osmylopsychopids, the abundant patterns unequivocally implied that the Daohugou osmylopsychopid species evolved the particular adaptations to the Jurassic environments. The Middle Jurassic Daohugou was considered to have a humid and warm climate with rich and diverse vegetations [17,18,19,20,24,25,26,27,28], and was deemed to be one of the important diversification centers of the Mesozoic insects [17]. The remarkable diversity of the Daohugou osmylopsychopids further supports this point, implying that the osmylopsychopids possibly experienced a large-scale and rapid radiation and evolution associated with the Middle Jurassic environments. Of course, more fossil specimens are needed to further help us understand the evolutionary process of osmylopsychopids in the future.

## Data Availability

The data presented in this study are available in the paper.
